# Efficacy of Poria Cocos Extract on Sleep Quality Enhancement: A Clinical Perspective with Implications for Functional Foods

**DOI:** 10.3390/nu15194242

**Published:** 2023-10-01

**Authors:** Hyeyun Kim, Heeyong Choi, Byong-Gon Park, Hyo-Jin Ju, Yeong-In Kim

**Affiliations:** 1 The Convergence Institute of Healthcare and Medical Science, Department of Neurology, Catholic Kwandong University, International St. Mary’s Hospital, Gangneung 22070, Republic of Korea; nuyikim@catholic.ac.kr; 2 College of Medicine, Catholic Kwandong University, International St. Mary’s Hospital, Incheon 22711, Republic of Korea; hy6753@naver.com (H.C.); fox9895@daum.net (H.-J.J.); 3 Department of Physiology, College of Medicine, Catholic Kwandong University, Gangneung 25601, Republic of Korea; bgpark@cku.ac.kr

**Keywords:** *Poria cocos*, insomnia, sleep, herbs

## Abstract

Background: Since the outbreak of the pandemic started, an increase in the number of sleep disorders, including insomnia and poor sleep quality, has been seen. The pattern will probably continue. Methods: This study focuses on the preparation and clinical testing of *Poria cocos* extract in treating suboptimal sleep quality. The optimal extraction method utilized a 75% ethanol concentration, and the clinical investigation involved subjects with defined poor sleep taking 800 mg of the extract nightly, assessed using the Sleep Questionnaire and polysomnography. The non-parametric Wilcoxon signed-rank test was used for statistical analysis due to the non-normal distribution of the collected data. Results: The study involved 21 insomnia sufferers with a mean age of 55 who were administered *Poria cocos* extracts. The findings of the study indicate a statistically significant rise in the overall duration of sleep (from 327.395 ± 43.2 min to 356.516 ± 63.21 min, *p* = 0.014). Additionally, there was a notable decrease in the level of arousal during sleep (from 76.316 ± 44.78 min to 47.989 ± 42.38 min, *p* = 0.009), and an improvement in the sleep severity index of the sleep questionnaire test. Conclusions: *Poria cocos* as a natural substance could improve quality of sleep, based on the findings. The study investigates Pachymic acid, a substance found in *Poria cocos*, as a potential indicator for the development of sleeping aids.

## 1. Background

According to a referenced research, it has been reported that the prevalence of sleep disorders in the general population is 87% [1]. Furthermore, the number of people who are reporting having trouble sleeping is fast increasing each year, which can be partially attributable to the pandemic of COVID-19. This pandemic has been sweeping the world [2]. When more conventional treatments, including alterations to one’s lifestyle, increased levels of physical activity, and controlled use of caffeine, are unsuccessful in treating chronic insomnia, individuals may want to consider the use of sleep-inducing drugs as an additional option. Herbal medicines are considered to be at the top of the list for sleep aids, followed by pharmaceuticals that are considered to be dietary supplements [3]. The very last thing that needs to be done is choosing a pharmaceutical substance that will make you sleepy. The worry that people have about becoming addicted to sleeping medications and the negative effects of doing so is the primary element that prompts them to go through cycles in which they use these medications. Insomnia medications typically have a rapid beginning of action, which is a benefit, but chronic use might have adverse effects if the medication is taken for an extended period of time. An excessive amount of use of sleeping pills may result in substantial consequences such as exhaustion, decreased focus and remembering, amplified sensitivity to falls among elderly people, and an increased risk of accidents. These results may be brought on by excessive use of sleeping pills. As a result, it is essential to make use of natural sleep aids and medicinal plants, both of which are devoid of any negative consequences [4].

The growing demand from society has resulted in the development of a potentially fruitful sub-business within the sleep health industry. This sub-industry involves the production of functional food products that are designed to facilitate sleep. Currently, there is an emphasis placed on herbs that have the possible ability to promote sleep, and, in Eastern regions, there is a re-evaluation of substances that are utilized in Eastern medicine as prospective food sources that promote sleep.

In the realm of forest products, several kinds of mushrooms stand out, owing to the fact that they have the potential to act as functional foods that improve both one’s health and one’s ability to obtain quality rest. In the food business and biopharmaceutical standards, fungi, and more specifically *Poria cocos*, as well as the Jannabi stool parasitic on the roots of pine trees, are recognized as an important component of natural medicine. The extract of *Poria cocos* has been the subject of a significant amount of research, which has led to the discovery that it possesses anti-tumor, anti-inflammatory, antioxidant, anti-aging, and cognitive function enhancement characteristics. The results of a number of separate research studies have been compiled to provide this body of evidence. It is well-known that *Poria cocos* is composed of a number of essential components, including Pachymic acid (C_33_H_52_O_5_), Pinylic acid (C_30_H_18_O_3_), Songnyeongsinic acid (3--Hyderoxy-lanosta-7.9(11), 24-Trien-21-oic acid, and Tumulosic acid (C_31_H_5_). [5] Additionally, poricoic acid and phosphoric acid (Cortoic acid (C_51_) are also derived from this source. The substance in question comprises perishaminoic acid, abricoic acid, polypothenic acid A and C, triterpenoid, ergosterol, lecithin, adenine, choline, glucose, fructose, protein, and a significant quantity of inorganic compounds. The presence of a pachymic acid concentration of 93% in *Poria cocos* that meet the necessary standards serves as a major criterion in this study regarding medications for insomnia. The investigation was conducted by the University of Hawaii. There is evidence that pachymic acid can cause a variety of effects, including the maintenance of cognitive and physical homeostasis, the promotion of mental equilibrium, and the augmentation of diuretic function [6,7,8]. The effectiveness of this medication has been established in situations of cognitive decline, insomnia, and chronic gastritis, in addition to cases of physical weariness and weakness. It is known for its capacity to promote the release of serotonin, which is a neurotransmitter in the brain. Serotonin has been demonstrated to alleviate symptoms of anxiousness, stress, and depression, as well as help with the prevention and treatment of insomnia. This property of the herb has earned it widespread recognition. According to the findings of the previous research, the investigation’s objective was to determine whether or not the extract of *Poria cocos* could improve the clinical performance of functional meals.

## 2. Methods 

### 2.1. Preparation of Poria cocos Extract 

The quantity of pachymic acid that is found in unprocessed mushroom specimens has been shown to have a positive link with the concentration of ethanol that is used as the extracting agent, according to research that had been conducted in the past. According to the findings of previous research, the extraction method that was used in this clinical investigation consisted of using a 75% concentration of ethanol for the synthesis of *Poria cocos* extract. Pachymic acid is a critical indicator chemical, and its optimal extraction time was discovered by optimizing the extraction process. The solvent used in the extraction process was ethanol with a concentration of 75%, and the extraction period ranged from one to eight hours. After conducting in-depth research, it was determined that an extraction duration of four hours produced the best results, with the peak area value being constant within a 10% range throughout the process. 

During the concentration step, the process of exchanging ethanol for water led to the production of a sizeable amount of suspended particles, which was a significant consequence. These solids began to attach to the walls of the flask as the concentration process continued for a longer period of time. Therefore, the cutoff point for the concentration was decided to be set at 6 brix, which is the threshold value at which wall adhesion begins to take place. Excipient dextrin was added to the extracts, and then those extracts were freeze-dried so that a comparative study of the respective behaviors of each excipient could be carried out. According to the results of the investigation, the freeze-dried specimen could not be retrieved because it had attached to the internal surfaces of the extraction vessel. On the other hand, the freeze-dried specimen revealed substantial hygroscopicity, which was a problem that could be fixed by adding 30% more excipient. Experimentation with the addition of various excipients and various drying methods were required for the study in order to produce either a powder or a concentrate. According to the findings, the most effective method was to begin by using dextrin, then go on to the freeze-drying process.

### 2.2. Proper Dosages of Poria cocos Extracts 

Table 1 shows the dosages and percentages of *Poria cocos* extracts utilized by the participants in the study. [9] The study protocol entailed oral administration of 2 capsules containing a total of 800 mg of the investigational *Poria cocos* extract to the participants, once daily, 30 min to 1 h prior to bedtime, accompanied by an ample amount of water. 

### 2.3. A Clinical Investigation Targeting Individuals with Suboptimal Sleep Quality 

Participants who exhibited suboptimal sleep quality were operationally defined as those who scored 5 or higher on the Pittsburgh Sleep Quality Questionnaire (PSQI) [10] and had an Insomnia Severity Index (ISI) score ranging from 8 to 21 [11]. The aforementioned condition was uniform across all participants, with the exception of individuals who had coexisting sleep disorders that may lead to insomnia, such as sleep apnea, narcolepsy, circadian rhythm disorders, event sleep, restless legs syndrome, and periodic limb movement disorder. The use of sleeping medicines or supplements that promote sleep by participants was one of the criteria that led to their exclusion from the study. Another exclusion criterion was the participants’ expectation that they would consume alcohol or smoke cigarettes during the course of the research. Participants in the study who were obliged to use medications, such as antihyperlipidemic drugs, bronchodilators, beta-blockers, or corticosteroids, were likewise excluded from the research. Every participant in the study had an initial polysomnography (PSG) and answered questions about their sleeping habits in a questionnaire. The individuals who took part in the study were instructed to take in 800 milligrams of *Poria cocos* extract products on a daily basis for the duration of the study, beginning exactly one hour before the time that they normally go to bed. After the treatment was finished, a post-treatment evaluation was carried out using a questionnaire and a PSG to determine how the use of *Poria cocos* extract had affected the participants.

### 2.4. Statistics

Given that the data gathered in this investigation fail to satisfy the prerequisites of normal distribution, the Wilcoxon signed-rank test, which is appropriate for non-parametric pre- and post-intervention comparisons, was employed to assess the efficacy and stability of the intervention. 

The Wilcoxon signed-rank test is a statistical analysis technique that involves ranking the absolute value of variable value differences pre- and post-experimentation, assigning the sign of the variable value difference to the calculated rank, and, subsequently, summing these ranks to determine statistical significance. 

## 3. Results

### 3.1. General Characteristics

In conclusion, there were twenty people with insomnia who participated. Everyone who took part in the study complained of having trouble sleeping, but none of them had ever tried any health-enhancing vitamins or prescription medications. A total of 21 participants were finally included in the analysis of the results. Three of them were men. The study’s findings indicate that the mean age of the participants was 55 years with a standard deviation of 19.80. The average height, weight, and BMI values were 161.48 cm ± 9.57, 64.24 kg ± 10.01, and 24.48 ± 2.37, respectively, as presented in Table 2.

### 3.2. The Results of Sleep Study including PSG and Sleep-Related Questionnaires

When compared to the measurement taken at the beginning of the study, the researchers discovered that the variable value of the ISI decreased after the administration of *Poria cocos* extracts, and this reduction was statistically significant. The PSQI evaluation revealed that the quality of sleep had improved significantly, as shown by a *p*-value of 0.05. This improvement was statistically significant. 

**Figure 1:**
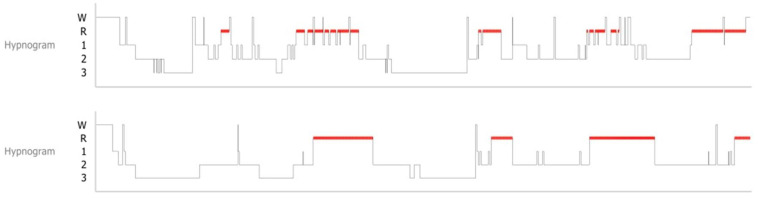
Sleep architecture changed from baseline hypnogram (**Upper**) to post-medication hypnogram (**Lower**). Sleep fragmentation improved after taking *Poria cocos* extracts, as did frequent awakening.

Table 3 showed a potential increase in sleep efficiency from 77.737% ± 9.72% to 81.584% ± 16.7% (*p* = 0.07). The study found a statistically significant increase in total sleep time (TST) from 327.395 ± 43.2 min to 356.516 ± 63.21 min, indicating an approximate increase of 30 min (*p* = 0.014). The percentage of time spent in rapid eye movement (REM) sleep during TST exhibited a statistically significant increase from 18.442% ± 4.68% to 22.053% ± 4.52% (*p* = 0.005). The study found a statistically significant reduction in the duration of waking after sleep onset (WASO) from an average of 76.316 ± 44.78 min to 47.989 ± 42.38 min (*p* = 0.009). The latency to REM exhibited a significant decrease from an average of 108 ± 52.67 min to 77 ± 52.98 min (*p* = 0.004). The study findings indicate a statistically significant decrease in ISI scores from 13 ± 5.75 to 10.55 ± 5.22 during the administration of the sleep questionnaire (*p* = 0.012), as presented in Table 2. Furthermore, it was observed that there was a rise in the length of REM sleep and a decline in the duration of time taken to transition from non-REM sleep to REM sleep, indicating an enhancement in the structure of sleep (Figure 1). The results of the blood tests conducted prior to and subsequent to the administration of *Poria cocos* extracts did not demonstrate any noteworthy alterations from the initial values. Table 4 presents the findings. The result of a serologic test showed a statistically significant decrease in the value of potassium after the experiment (*p* = 0.041).

## 4. Discussion

*Poria cocos* is a well-known traditional Eastern medicine that can be found in the soil close to the roots of pine trees in China, Korea, Japan, and North America. In Asia, *Poria cocos* has been used either alone or in combination with other herbs to alleviate insomnia [12]. Experimental research has amply confirmed a number of *Poria cocos*’ pharmacological properties [13]. *Poria cocos* is a traditional Oriental medicinal material known to have anti-inflammatory activity, immunomodulatory properties, and anticancer, antioxidant, and antihyperglycemic effects [13,14,15]. The researchers, among other things, carried out tests on animals, as well as clinical research, with the hope that the GABAergic action would lead to improvements in sleep quality. Refs. [16,17] Based on the mechanism of the GABAergic effect, the main indicator substance of *Poria cocos* extract was pachymic acid, and a 75% ethanol extraction method was selected to extract a high concentration of pachymic acid. We have preliminary research to support the 75% ethanol extraction. Patients with insomnia generally report having trouble falling asleep, staying asleep, or having poor quality sleep. Insomnia is a very common illness that is described as perceived inadequate sleep [18]. Targeting Gamma-Aminobutyric Acid (GABA)_A_ receptors, which are the main mediators of rapid inhibitory neurotransmission in the central nervous system, sedative-hypnotics are frequently used to treat insomnia and reduce anxiety levels [19]. The inhibitory neurotransmitter GABA activates these receptors, which modify the performance of chloride ion channels [20]. Controlling neurotransmitters in the central nervous system, such as serotonin or the GABAergic systems, has been proven to be the mechanism by which a number of plants are able to induce sedative and hypnotic effects [21]. According to research, benzodiazepines can affect a variety of brain rhythmic processes by inhibiting the release of the inhibitory neurotransmitter GABA. Pachymic acid from the ethanol extract of *Poria cocos*, which is the main target in this study, alters sleep patterns by stimulating GABA-ergic systems [22]. In this study, they were able to successfully promote health booster supplements using *Poria cocos* ethanol extract, as well as effectively conduct a clinical trial using it, and report the findings. After using the trial product containing *Poria cocos* extracts, participants who were experiencing symptoms of insomnia reported improvements in both the duration of their sleep and the quality of their subjective sleep. The most important thing that was accomplished by the study was providing objective data on the clinical efficacy of *Poria cocos* extracts. This was accomplished by demonstrating a rise in TST, an increase in sleep efficiency, and a decrease in WASO based on the results of the PSG. As can be seen in Figure 1 of the findings of the study, in addition to an improvement in the quantitative polysomnographic values, we also noted a normalization of the general architecture of sleep. This was observed to go along with the improvement. When it was returned to its usual state and WASO scored much higher, patients in clinical trials also reported subjectively experiencing a considerable improvement in the quality of their sleep. It is noteworthy that these changes in structure occurred after only two weeks of treatment, and is the most important favorable effect that the trial medication achieved. Because of this, it is more common for health-promoting supplements to be ineffective or require an extended period of treatment than sleep pills recommended by a physician. The explanation for this is due to the fact that the body reacts differently to any one of them. Even though the results were observed after only two weeks of usage, several participants reported experiencing subjective improvements in sleep quality after only two to three days of use; hence, a suitable duration of the administration of *Poria cocos* extract needs to be further investigated.

In order to verify that the *Poria cocos* extract was free of any harmful hematological side effects, we put it through a series of tests both before and after it was administered. However, there were only a small number of people who volunteered to be examined (as shown in Table 4), which makes it challenging to make generalizations of the findings. In the results of the tests, as such, we were able to observe a drop in the level of serum potassium. Even though this is a shift that can be considered statistically significant, it is still considered to be within the normal range; thus, any interpretations of it should be made with the utmost caution. Despite this, these findings need to be taken seriously and verified in additional safety research.

A general increase in the number of people who are unable to obtain adequate sleep has occurred since the start of the COVID-19 epidemic, which was characterized by increased social isolation and a general decrease in the amount of activities that took place outside. This fact was mentioned recently. In an effort to lessen the detrimental impacts of insomnia, research is always being performed on a variety of interventions, including prescription sleep medications and cognitive behavioral therapy for insomnia. Due to prevalent side effects such as somnolence throughout the day, falling frequently, and decreased focus due to using night sleep drugs, treating patients who are elderly, female, or teenagers can be particularly challenging for physicians. Therefore, choosing the right prescription at the proper dosage and for a suitable amount of a period of time, as well as choosing the optimal sleeping pill, given its psychopharmacologic consequences, is a common challenge for physicians. The medical profession as a whole and individual patients are looking for pharmacological solutions for sleep that are less addictive and result in fewer undesirable side effects than the options that are now advised by physicians. These new options would also be preferable. The widespread prevalence of insomnia in both the East and the West has led to the discovery of numerous herbs that have been shown to be capable of assisting in the process of inducing sleep. An equally large number of nutritional supplements make use of these plant-based products. On the other hand, clinical research on health-promoting supplements has been carried out on a smaller scale in comparison to clinical studies on sleep medications, which means that there is a lack of independent proof of effectiveness that is now available.

We developed a health supplement that aids in sleep by utilizing an extract from *Poria cocos*, and we based the dosage for clinical patients on research conducted on animals. We were successful in creating the dietary supplement by making use of all of the aforementioned data. On the basis of this, we decided to conduct a clinical trial, and we were able to validate the results by conducting PSG in order to show that there was an objective improvement in sleep quality. Because these extracts have been proven to improve the quality of sleep at night for individuals who suffer from poor sleep through a GABAergic mechanism, we would want to discuss the possibility of using them as a component in the development of new sleep drugs. This is because we would like to explore the possibility of using them as a component in the creation of new sleep medications. Following the completion of additional research, we have decided to propose that this medicine be used as a sleep pill.

This study has several limitations. First, because there are many different mechanisms and pathophysiologies of insomnia, there were no controls for age, gender, sleep environments, or working types. Due to the small number of participants in the final study, subgroup analysis was not conducted. Future studies should include subgroup studies based on different mechanisms of insomnia. Further research could identify the therapeutic target age and gender for health-promoting foods using *Poria cocos* extract. Second, the duration and dosage of *Poria cocos* extract has been tested in animals, but clinical trials with different durations and dosages have not been conducted in this study, so it is not possible to provide a final recommended dose of *Poria cocos* extract to improve insomnia. This should be supplemented by further research. Third, we developed this product with a focus on pachymic acid, but lacked consideration of the various other active ingredients in *Poria cocos*. Further research is needed to validate the various active ingredients. This would be a crucial second step in the direction of our end goal, which is to use *Poria cocos* to develop a novel drug for the treatment of insomnia.

## 5. Conclusions

Researchers are of the opinion that extracts obtained from *Poria cocos* have the potential to function as a new category of sleep aid in the not-too-distant future. It is anticipated that more study efforts will give discoveries regarding the amounts of pachymic acid and its precise pharmacological mechanism in promoting sleep, derived from *Poria cocos*, with greater effectiveness. This is due to the fact that additional research initiatives will be undertaken. 

## Figures and Tables

**Table 1:** Ingredient names and formulation ratios (%), *Poria cocos* (800 mg).

Ingredient Names	Formulation Ratios (%)	Contents (mg)	Intake per Day (mg)
*Poria cocos* extracts	75.47	400.00	800.00
Cornstarch (Starcap)	21.83	115.69	231.38
Silicon dioxide	1.70	9.01	18.02
Magnesium stearate	1.00	5.30	10.60
Total	100.00	530.00	1060.00

**Table 2:** General characteristics of all participants (*N* = 21).

	Mean ± SD	Min.	Max
Age (*N* = 21)	55 ± 19.80	24.0	86.0
Height (cm)	161.48 ± 9.57	144.0	175.0
Weight (kg)	64.24 ± 10.01	48.0	85.0
BMI	24.48 ± 2.37	21.1	31.2

BMI, body mass index.

**Table 3:** The results of polysomnography and questionnaires associated with sleep.

	Pre	Post	z	*p*
	Mean ± SD			
TIB	401.9 ± 73.73	421.711 ± 36.16	−0.806 ^b^	0.42
TST	327.395 ± 43.2	356.516 ± 63.21	−2.455 ^b^	0.014
N1 (% of TST)	14.695 ± 6.46	11.863 ± 4.36	−1.731 ^c^	0.083
N2 (% of TST)	43.695 ± 10.64	43.411 ± 6.1	−0.081 ^c^	0.936
N3 (% of TST)	28.142 ± 22.15	22.663 ± 8.28	−1.167 ^c^	0.243
REM (% of TST)	18.442 ± 4.68	22.053 ± 4.52	−2.818 ^b^	0.005 *
WASO	76.316 ± 44.78	47.989 ± 42.38	−2.616 ^c^	0.009 *
Latency to REM	108 ± 52.67	77 ± 52.98	−2.860 ^c^	0.004 *
Sleep efficiency	77.737 ± 9.72	81.584 ± 16.7	−1.811 ^b^	0.07
AHI	12.026 ± 7.36	11.658 ± 7.26	−0.725 ^c^	0.469
PLMS index	6.126 ± 10.55	7.121 ± 13.49	−0.280 ^b^	0.779
Desaturation Index	85.784 ± 8.05	86.474 ± 3.87	−0.423 ^c^	0.672
PSQI	8.095 ± 3.76	6.3 ± 2.97	−1.940 ^c^	0.052 *
ISI	13 ± 5.75	10.55 ± 5.22	−2.524 ^c^	0.012 *

TIB, time in bed; TST, total sleep time; N, NREM; REM, rapid eye movement; WASO, wakefulness after sleep onset; AHI, Apnea Hypopnea Index; PLMS, periodic limb movement during sleep; PSQI, Pittsburgh Sleep Quality Questionnaire; ISI, Insomnia Severity Index; * Indicates statistical significance with *p* < 0.05. ^b^ after > before, ^c^ before > after.

**Table 4:** The results of serologic test.

	Pre	Post	z	*p*
	Mean ± SD			
RBC count	4.52 ± 0.5	4.47 ± 0.64	−0.384 ^b^	0.701
Hb	21.83 ± 31.36	13.23 ± 1.82	−1.575 ^b^	0.115
PLT count	353.53 ± 386.89	236.31 ± 41.97	−1.294 ^b^	0.196
WBC count	76.6 ± 274.54	5.28 ± 1.24	−0.874 ^b^	0.382
Protein (T)	7.29 ± 0.4	7.05 ± 0.27	−1.827 ^b^	0.068
Albumin	4.31 ± 0.25	4.16 ± 0.19	−1.429 ^b^	0.153
Total bilirubin	0.81 ± 0.37	8.42 ± 27.82	−0.360 ^c^	0.719
AST	31.07 ± 8.68	28.46 ± 8.75	−1.120 ^b^	0.263
ALT	31.27 ± 11.44	25.54 ± 14.79	−1.868 ^c^	0.062
Total cholesterol	182.27 ± 42.14	185.15 ± 49	−0.665 ^c^	0.506
BUN	15.53 ± 2.76	15.48 ± 3.75	−0.175 ^b^	0.861
Creatinine	0.71 ± 0.15	0.73 ± 0.12	−0.629 ^c^	0.529
Uric acid	4.81 ± 0.93	5 ± 1.11	−0.906 ^c^	0.365
Na (Sodium)	139.4 ± 1.59	130 ± 34.91	−0.142 ^b^	0.887
K (Potassium)	4.3 ± 0.27	4.15 ± 0.31	−2.047 ^b^	0.041 *
Cl (Chloride)	104.8 ± 1.37	98.15 ± 25.09	-0.136 ^b^	0.892
Glucose	106.73 ± 21.28	122.08 ± 32.22	−1.784 ^c^	0.074
P (Phosphorus)	32.29 ± 111.13	3.72 ± 0.65	−0.105 ^c^	0.916
Ca (Calcium)	9.76 ± 0.64	9.56 ± 0.23	−0.039 ^c^	0.969
Mg (Magnesium)	2.04 ± 0.13	2.04 ± 0.14	−0.250 ^b^	0.803

* Indicates statistical significance with *p* < 0.05. ^b^ after > before, ^c^ before > after.

## Data Availability

Not applicable.

## Abbreviations

GABA	Gamma-aminobutyric acid
ISI	Insomnia Severity Index
PSG	Polysomnography
PSQI	Pittsburgh Sleep Quality Index
REM	Rapid eye movement
TST	Total sleep time
WASO	Waking after sleep onset
